# An investigation of patterns in hemodynamic data indicative of impending hypotension in intensive care

**DOI:** 10.1186/1475-925X-9-62

**Published:** 2010-10-25

**Authors:** Joon Lee, Roger G Mark

**Affiliations:** 1 Harvard-MIT Division of Health Sciences and Technology, 45 Carleton Street, Cambridge, MA 02142, USA; 2 Massachusetts Institute of Technology, 77 Massachusetts Avenue, Cambridge, MA 02139, USA

## Abstract

**Background:**

In the intensive care unit (ICU), clinical staff must stay vigilant to promptly detect and treat hypotensive episodes (HEs). Given the stressful context of busy ICUs, an automated hypotensive risk stratifier can help ICU clinicians focus care and resources by prospectively identifying patients at increased risk of impending HEs. The objective of this study was to investigate the possible existence of discriminatory patterns in hemodynamic data that can be indicative of future hypotensive risk.

**Methods:**

Given the complexity and heterogeneity of ICU data, a machine learning approach was used in this study. Time series of minute-by-minute measures of mean arterial blood pressure, heart rate, pulse pressure, and relative cardiac output from 1,311 records from the MIMIC II Database were used. An HE was defined as a 30-minute period during which the mean arterial pressure was below 60 mmHg for at least 90% of the time. Features extracted from the hemodynamic data during an observation period of either 30 or 60 minutes were analyzed to predict the occurrence of HEs 1 or 2 hours into the future. Artificial neural networks (ANNs) were trained for binary classification (normotensive vs. hypotensive) and regression (estimation of future mean blood pressure).

**Results:**

The ANNs were successfully trained to discriminate patterns in the multidimensional hemodynamic data that were predictive of future HEs. The best overall binary classification performance resulted in a mean area under ROC curve of 0.918, a sensitivity of 0.826, and a specificity of 0.859. Predicting further into the future resulted in poorer performance, whereas observation duration minimally affected performance. The low prevalence of HEs led to poor positive predictive values. In regression, the best mean absolute error was 9.67%.

**Conclusions:**

The promising pattern recognition performance demonstrates the existence of discriminatory patterns in hemodynamic data that can indicate impending hypotension. The poor PPVs discourage a direct HE predictor, but a hypotensive risk stratifier based on the pattern recognition algorithms of this study would be of significant clinical value in busy ICU environments.

## Background

In the intensive care unit (ICU), hypotension is a critical condition that requires prompt therapeutic intervention. Persisting hypotension can result in dangerously decreased tissue blood flow with consequent end-organ damage. As a result, ICU clinicians must be vigilant to detect and treat hypotensive episodes (HEs) in a timely manner. However, this is challenging to achieve for several reasons. First, the amount of time that clinical staff can allocate per patient is generally limited, particularly in ICUs facing staff shortages - a problem that is projected to get worse in the future [1]. Second, the amount of physiologic and clinical data that is accumulated per patient is enormous and growing. The resultant "data overload" has significantly complicated patient assessment [2]. ICU data is not only massive in size but is also heterogeneous in nature due to their vastly different sources (multi-channel waveforms, laboratory results, medication records, nursing notes, etc.) and suboptimal organization. Third, even with sufficient time, resources, and information, it seems extremely difficult to estimate the likelihood of an impending HE with bare-eye analysis alone, especially for HEs that are not preceded by obvious patterns such as a gradual decrease in blood pressure.

Hence, in the stressful context of busy ICUs, it clearly would be of considerable clinical value to *prospectively *identify patients who are at risk of developing HEs in the next one to two hours, since it would facilitate efficient allocation of ICU resources and minimize the time delay to appropriate therapy. Such prospective hypotensive risk stratification would not necessarily mandate immediate clinical action, but rather function as a screening test to identify a subgroup of patients at risk of hypotension so that clinicians can focus vigilance and care. The resulting preparedness would ensure prompt therapeutic intervention. The efficacy of prompt initiation of appropriate therapy has been shown with severe sepsis [3], shock [4], and acute coronary syndrome [5].

Hemodynamic instability with the potential to lead to an HE may be detectable by sophisticated analysis of routinely monitored physiologic data, since they might reflect the underlying dynamics of the cardiovascular neurohumoral controls reacting to pathological stress. Continuous and quantitative analysis of complex medical data is a suitable task for a computer in comparison with a human clinician. In particular, multi-parameter time series of physiologic variables may contain subtle patterns that are a signature of impending frank hemodynamic instability, and such patterns are best identified and characterized by machine learning algorithms. Real-time advance alerts can change ICU care from "reactive" to "proactive" [6].

There has been limited research at the intersection between pattern recognition and hypotension analysis. Among the few past research efforts, one approach utilized a wavelet-based similarity measure and time-series retrieval to yield an area under receiver operating characteristic curve (AUC) of 0.83 in predicting vasopressor onsets [7,8]. The recent PhysioNet Challenge on hypotension prediction was successful in motivating related research, and the winner scored an accuracy of 93% in a test data set of 40 patients [9]. The importance of automated or semi-automated assistance in analyzing multimodal ICU data is also increasingly recognized [10].

As a first step toward an automated hypotensive risk stratifier, the main objective of the current study was to investigate the possible existence of discriminatory patterns in ICU data that can distinguish impending hypotensive episodes from normotension. To conduct the investigation, artificial neural networks were deployed to find the discriminatory patterns, in both binary classification (hypotensive vs. normotensive) and regression (blood pressure estimation). We anticipate that the results reported in this paper will be of interest to biomedical engineers interested in developing decision support systems as well as to ICU clinicians.

## Methods

### Data compilation

A subset of the Multi-parameter Intelligent Monitoring for Intensive Care (MIMIC) II database [2] was analyzed in this study. For details about the publicly available MIMIC II database, please refer to its documentation [11]. The time series data in the MIMIC II database are organized into records, each of which corresponds to a particular ICU stay. The records that met the following inclusion criteria comprised the subset:

• The record was from an adult patient.

• Minute-by-minute time series of heart rate (HR) and systolic (SBP), diastolic (DBP), and mean arterial blood pressure (MAP) computed by bedside monitors were present.

• Age and medication information, which is part of the clinical data in the MIMIC II database, was present.

• Hemodynamic time series and clinical data had been matched (i.e., it was certain that the time series and clinical data had come from the same ICU stay of the same patient).

**Figure 1:**
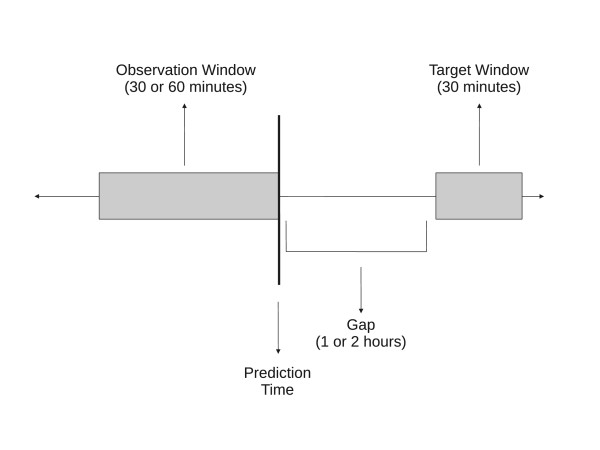
**A graphical illustration of the observation window, gap, and target window with respect to prediction time**.

From each selected record, one or more examples were compiled, depending on the compilation mode (see below for more details). Each example consisted of three time intervals: (1) an *observation window *of either 30 or 60 minutes; (2) a *target window *of 30 minutes; and (3) a *gap *of either 1 or 2 hours separating the observation and target windows. The information in the observation window served as the basis of the inputs to the pattern classifiers and a prediction was generated at the end of the observation window. This setup is graphically illustrated in Figure 1. Different observation window sizes were analyzed to see if observing the time series for a longer period time would improve prediction, whereas different gap sizes investigated whether predicting further into the future corresponds to a more challenging problem. 

Each target window was labeled either "control" or "hypotensive". Although there are a number of possible definitions of HE, we arbitrarily defined an HE as a 30 minute period in which MAP was less than 60 mmHg and greater than 10 mmHg for at least 90% of the 30 minute period. Any 30 minute window that did not meet the HE definition and contained MAP values between 10 and 200 mmHg for more than 90% of the window was regarded as a control (normotensive) example. The lower and upper bounds of 10 and 200 mmHg served as a crude filter that eliminated physiologically unlikely outliers. Examples with target windows that satisfied neither definition were excluded from the study. For each target window, the corresponding observation window was also checked for physiologic validity; all of the 4 time series (HR and 3 arterial blood pressure (ABP)) in the observation window must have contained values between 10 and 200 (in mmHg and bpm for ABP and HR, respectively) for more than 95% of the window. All examples with observation windows that failed to pass this validity check were excluded from analysis. 

In addition, there were two example compilation modes: *single *and *multiple*. In single compilation, only one example was obtained from each record. Because there were far fewer hypotensive than control examples, each record was checked for a hypotensive example first. Control examples were compiled from the records that contained no hypotensive episode. Since each record almost always contained multiple control or hypotensive examples, one example was randomly selected, with equal probabilities, to be included in the data. In multiple compilation, on the other hand, a 30 minute sliding window with no overlap traversed each record, and as many examples as possible were compiled, provided that the validity checks for the observation and target windows were passed.

### Feature extraction

For feature extraction, two additional time series were derived as follows:

• Pulse pressure (PP) was derived using *PP *= *SBP *- *DBP*.

• Relative cardiac output (CO) was estimated using *CO *= *HR *× *PP *according to the Windkessel model [12]. This estimation differs from the actual cardiac output by a scalar multiple, equal to arterial compliance, but this relative CO was sufficient for the purpose of pattern recognition. Sun *et al*. [13] evaluated this Windkessel estimator against thermodilution CO measurements and reported that its estimation error was lower than several more advanced CO estimation methods, despite its crude nature.

Based on all 6 time series (HR, SBP, DBP, MAP, PP, CO) and clinical data, a total of 102 features were extracted from the observation window of each example. The strategy was to rely on pattern recognition tools for finding discriminatory information in the time series without any *a priori *knowledge. Thus, it was crucial that a wide selection of features were included to capture various signal aspects and hence to maximize the likelihood of finding discriminatory information.

The features were loosely classified into 4 groups: statistical, wavelet, cross-correlation, and clinical features. These feature groups are described in the ensuing sections.

#### Statistical features

Mean, median, standard deviation, variance, interquartile range, skewness, kurtosis, and linear regression slope were computed for each of the 6 time series. Mean and median quantitatively represent the magnitude of each physiologic variable, whereas standard deviation, variance, and interquartile range describe its variability. Variability may be indicative of the effort of the cardiovascular system to control blood pressure and of the related hemodynamic instability [14]. Skewness and kurtosis are the third and fourth moments of amplitude distribution, respectively, and reflect the shape of the distribution. Lastly, linear regression slope was calculated using the least-squares criterion and measures the average rate of change in the observation window.

#### Cross-correlation features

The cross-correlation at zero lag, which is a measure of coupling between two time series, was computed for all possible time series pairs. If two distinct time series are denoted as *X *= {*x*_1, _*x*_2_, ..., *x_n_*} and *Y *= {*y*_1_, *y*_2_, ..., *y_n_*}, the cross-correlation between them was computed as follows:

(1) $$R_{XY}(0)=\frac{1}{n}\sum_{i=1}^{n}x_{i}y_{i}$$

#### Wavelet features

To capture relative energies in different spectral bands, a 5-level discrete wavelet decomposition of each of the 6 time series was conducted with the Meyer wavelet. Such a wavelet decomposition breaks down the variability of a given time series into different spectral bands. If the decomposition for a given time series *X *is denoted as *W_X _*= [*a*_5 _*d*_5 _*d*_4 _... *d*_1_], where *a*_5 _is the approximation signal and *d_k _*is the *k^th^*-level detail signal, the energy in *a*_5 _was computed as follows:

(2) $$E_{a_{5}}=||a_{5}|{|}^{2}$$

where ||•|| is the Euclidean norm. Similarly, the energy in the *k^th^*-level detail signal was:

(3) $$E_{d_{k}}=||d_{k}|{|}^{2}$$

for *k *= 1, 2, ..., 5. Finally, the relative energy contribution from each decomposition level was calculated as follows:

(4) $$Er_{a_{5}}=\frac{E_{a_{5}}}{E_{T}}$$

(5) $$Er_{d_{k}}=\frac{E_{d_{k}}}{E_{T}}$$

for *k *= 1, 2, ..., 5 and where

(6) $$E_{T}=E_{a_{5}}+\sum_{k=1}^{5}E_{d_{k}}$$

These wavelet energy features have been frequently applied in a variety of signal processing and pattern recognition studies (e.g., [15-17]).

#### Clinical features

Patient age was utilized as a feature to examine if certain age groups are more likely to develop HEs. Since the same age was recorded for all examples from the same patient, it only provided an *a priori *bias, rather than temporally localized information.

Also, the amounts of hemodynamically active medications, in mcg/kg, delivered to the patient during the observation window were computed. Such medications influence blood pressure, and logically, their administration should impact short-term blood pressure level. Two types of medications were considered as separate features: 1) vasoconstrictors and positive inotropic drugs which tend to raise blood pressure, and 2) vasodilators, diuretics, and sedatives which tend to decrease blood pressure. The first group included the following medications: neosynephrine, norepinephrine, vasopressin, dopamine, dobutamine, epinephrine, milrinone, and isuprel. The following medications comprised the second group: nitroglycerine, nitroprusside, diltiazem, esmolol, labetalol, and lasix.

### Dimensionality reduction

Each extracted feature was first normalized to be zero-mean and unit-variance. Subsequently, the raw feature dimensionality of 102 was reduced via principal component analysis (PCA). PCA is a popular, widely-known dimensionality reduction algorithm that has been employed in previous pattern recognition applications in biomedical engineering, including prosthetic control based on the myoelectric signal [18] and classification of gene expression microarray data [19]. In this study, PCA was executed on training data and retained the principal components with the largest eigenvalues that captured approximately 90% of the total variance. Both training and test data were projected onto the same feature space defined by the selected principal components. Across different training data sets in cross-validation (the details of the cross-validation are to be discussed in the next section), the reduced dimensionality ranged from 15 to 19. This substantial reduction in dimensionality implies that PCA removed redundancy among the features. The transformed features in reduced dimensionality served as the inputs to classification and regression models, which are described next.

### Classification

According to the label assigned to each example (control or hypotensive), artificial neural networks were trained to perform a binary classification. Independent neural networks were trained for different combinations of gap and observation window sizes, as well as for different cross-validation folds and compilation modes. In both compilation modes, a 5-fold cross-validation was conducted to evaluate classification performance. However, in order to balance the two groups in training data so that the classifier is prevented from favoring the majority group, a subset of the majority group (which was always the control group) was randomly sampled without replacement. This randomized sub-sampling was repeated 10 times. On the other hand, test data were left unbalanced. Moreover, the partition between training and test data was conducted with respect to records rather than individual examples. In other words, examples from the same record belonged exclusively to either training or test data. The assumption here was that examples from the same record, which could be immediately next to one another, are likely to contain similar patterns. Then, once trained on several examples, other examples from the same record may be easier to classify, which would lead to over-estimated classification performance.

Feed-forward, 3-layer neural networks with one hidden layer of 20 hidden units were trained. The neural networks utilized the log-sigmoid activation function in both the hidden and output layers. Because the log-sigmoid activation function is bounded between 0 and 1, it is an appropriate choice for outputs in probability, which is the case in this binary classification. Also, it has been proven that this 3-layer architecture with sigmoid activation functions can approximately realize any continuous input-output mapping [20]. The threshold on the posterior probability was determined from the classifier's receiver operating characteristic (ROC) curve based on training data. The selection criterion for the optimal threshold was the following:

(7) $$Th_{s}=\underset{Th}{\arg\max}\{sensitivity(Th)+specificity(Th)\}$$

where *Th_s _*is the selected threshold and *Th *is the threshold variable ranging from 0 to 1. A random 20% partition of the training data was utilized for validation. During training, early stopping based on the validation set was employed for regularization.

For performance evaluation, AUC, accuracy, sensitivity, specificity, positive predictive value (PPV), and negative predictive value (NPV) were calculated.

### Regression

To investigate the feasibility of directly estimating the MAP in the target window, a regression approach was also pursued. The same neural network architecture as that in classification was employed, except that the activation functions were the hyperbolic tangent sigmoid and linear function in the hidden and output layers, respectively. This combination of activation functions ensures a dynamic swing on the output that is necessary for regression. Since the binary labels are irrelevant to regression, the distinction between the control and hypotensive groups was eliminated. Instead, the blood pressure in each target window was represented by the median MAP in the window. A 5-fold cross-validation was employed again; however, due to the large number of examples in multiple compilation (over 141,000), a subset of 2,500 examples was randomly constructed without replacement as training data. This randomized sub-sampling was repeated 10 times. As in classification, the inclusion of examples from the same record in both training and test data was prohibited.

Regression performance was evaluated by computing a mean absolute error, *MAE*, which was computed as follows:

(8) $$MAE\;=\frac{1}{N}\sum_{i=1}^{N}\frac{\left|{\hat{y}}_{i}-y_{i}\right|}{y_{i}}\times 100\%$$

where *N *is the total number of test cases, ${\hat{y}}_{i}$ is the *i*th predicted MAP, and *y_i _*is the corresponding true MAP. Furthermore, a linear regression line was fit to the scatter plot of true versus predicted MAP with the least-squares criterion. The least squares were iteratively reweighted with a bisquare weighting function. The slope and intercept of the regression line were then reported as performance metrics. In perfect regression, the slope and intercept should be 1 and 0, respectively. Lastly, the Pearson correlation coefficient between the predicted and true blood pressure was computed.

## Results

### Data compilation

After applying the inclusion criteria, a total of 1,311 records were compiled from MIMIC II for analysis. The median duration of the records included in this study was 79.8 hours with an interquartile range of 100.2 hours (*Q*_1 _= 41.7 h, *Q*_3 _= 141.9 h).

The exact numbers of compiled examples for the control and hypotensive groups were dependent on gap and observation window sizes as well as compilation mode. In single compilation, the number of examples ranged from 490 to 542 for the control group and from 317 to 333 for the hypotensive group. In multiple compilation, it varied from 129,853 to 137,753 and from 3,460 to 3,652 for the control and hypotensive groups, respectively.

### Classification

Tables 1 and 2 tabulate the classification results from the single and multiple compilation modes, respectively. Several observations stand out. First, overall performance is much superior with multiple compilation. For instance, mean AUCs are roughly 10% greater in multiple than in single compilation. Second, changes in observation window size did not result in appreciable differences in performance. Third, the increase in gap size from 1 to 2 hours caused a decrease in almost every performance measure. This is an expected result since predicting farther into the future is intuitively a more challenging problem.  Fourth, sensitivities and specificities seem fairly balanced, which proves the efficacy of the balancing via sub-sampling. A balance between sensitivity and specificity would not naturally be achieved without such a balancing mechanism in strongly unbalanced data sets like the one from multiple compilation. Fifth, the PPVs in Table 2 are barely over 10%, whereas those in Table 1 are over 60%. This is due to the fact that the mismatch between the hypotensive and normotensive classes was much more prominent in multiple than single compilation.

**Table 1:** Classification performance with single data compilation (mean ± SD)

	Gap = 1 h		Gap = 2 h	
	ObsWin = 0.5 h	ObsWin = 1 h	ObsWin = 0.5 h	ObsWin = 1 h
AUC	0.809 ± 0.042	0.819 ± 0.029	0.786 ± 0.014	0.787 ± 0.047
Accuracy	0.737 ± 0.026	0.758 ± 0.032	0.713 ± 0.007	0.714 ± 0.040
Sensitivity	0.745 ± 0.053	0.748 ± 0.064	0.743 ± 0.041	0.755 ± 0.025
Specificity	0.732 ± 0.036	0.764 ± 0.032	0.694 ± 0.025	0.688 ± 0.051
PPV	0.633 ± 0.030	0.665 ± 0.039	0.614 ± 0.010	0.606 ± 0.050
NPV	0.826 ± 0.031	0.833 ± 0.037	0.810 ± 0.019	0.819 ± 0.024

**Table 2:** Classification performance with multiple data compilation (mean ± SD)

	Gap = 1 h		Gap = 2 h	
	ObsWin = 0.5 h	ObsWin = 1 h	ObsWin = 0.5 h	ObsWin = 1 h
AUC	0.914 ± 0.018	0.918 ± 0.015	0.890 ± 0.019	0.894 ± 0.019
Accuracy	0.863 ± 0.014	0.858 ± 0.016	0.827 ± 0.021	0.832 ± 0.019
Sensitivity	0.812 ± 0.030	0.826 ± 0.026	0.788 ± 0.044	0.794 ± 0.036
Specificity	0.864 ± 0.014	0.859 ± 0.016	0.828 ± 0.022	0.833 ± 0.019
PPV	0.138 ± 0.012	0.136 ± 0.013	0.111 ± 0.014	0.114 ± 0.009
NPV	0.994 ± 0.002	0.995 ± 0.001	0.993 ± 0.002	0.993 ± 0.002

### Regression

Tables 3 and 4 report the regression results from single and multiple compilation, respectively. Similar to the classification results in Tables 1 and 2, multiple compilation is associated with better regression performance than single compilation. Also, the increase in gap size from 1 to 2 hours induced worse performance, whereas observation window size had minimal impact on performance.

**Table 3:** Regression performance with single data compilation (mean ± SD)

	Gap = 1 h		Gap = 2 h	
	ObsWin = 0.5 h	ObsWin = 1 h	ObsWin = 0.5 h	ObsWin = 1 h
MAE (%)	16.40 ± 1.62	16.16 ± 1.54	16.67 ± 1.73	17.57 ± 2.76
CorrCoeff	0.628 ± 0.095	0.604 ± 0.075	0.604 ± 0.051	0.574 ± 0.109
LR Slope	0.888 ± 0.219	0.901 ± 0.140	0.859 ± 0.130	0.746 ± 0.171
LR Int (mmHg)	8.11 ± 16.41	5.98 ± 10.37	10.92 ± 9.93	19.55 ± 12.52

**Table 4:** Regression performance with multiple data compilation (mean ± SD)

	Gap = 1 h		Gap = 2 h	
	ObsWin = 0.5 h	ObsWin = 1 h	ObsWin = 0.5 h	ObsWin = 1 h
MAE (%)	9.86 ± 0.31	9.67 ± 0.31	11.00 ± 0.27	10.67 ± 0.35
CorrCoeff	0.721 ± 0.026	0.730 ± 0.012	0.660 ± 0.038	0.683 ± 0.030
LR Slope	0.996 ± 0.029	0.985 ± 0.020	0.979 ± 0.025	0.960 ± 0.033
LR Int (mmHg)	0.13 ± 2.47	1.08 ± 1.59	0.97 ± 1.90	2.82 ± 2.34

The mean MAEs in Table 4 are roughly 10%, which translates into a deviation from true MAP by no more than 10 mmHg since MAP is usually below 100 mmHg. Furthermore, the linear regression slopes and intercepts in Table 4 are quite close to the ideal values of 1 and 0, respectively, although the moderate correlation coefficients reveal that there is non-trivial variance in the scatter plot.

## Discussion

### HE Definition

The HE definition employed in this study is comprised of two parameters: the 60 mmHg threshold and 30 minute duration. However, there are many possibilities for these parameters, which makes the particular choices arbitrary. Although the threshold of 60 mmHg is an accepted clinical guideline [21] and has often been used in previous studies (e.g., [22,23]), other threshold values, usually ranging from 65 to 75 mmHg, have also been utilized (e.g., [24,25]). Physiologically, thresholds between 60 and 65 mmHg seem to be appropriate since the autoregulation of blood flow to vital organs is known to cease in that MAP range [26]. Also, while the 30 minute duration has been used as an HE threshold before (e.g., [27]), durations as short as 1 minute (e.g., [28]) and as long as 1 hour (e.g., [22]) have also been employed. One of the reasons for this variability is that the two parameters in the definition depend on each other. For example, a duration of mere 5 minutes would be detrimental when MAP is only 20 mmHg but perhaps not when it is 50 mmHg. In pattern classification paradigms, however, a threshold is a necessity because the distinction between hypotensive and normotensive examples is required for training. Given that it is impossible to find a perfect HE definition that would make the optimal decision for all cases, the task became selecting a reasonable definition instead. We believe that the HE definition in this study is a good compromise among the possible definitions.

Moreover, a related issue is that the binary classification approach required a hard class membership, and performance evaluation on borderline examples may have been too strict. For instance, a target window that consistently exhibits MAP between 60 and 65 mmHg would be a control example in this study. If the classifier predicts an HE for this target window, the validity of counting this as a false positive may be questionable, especially from a clinical perspective. In fact, the primary purpose of the regression approach was precisely to circumvent this issue. Regression avoids the borderline problems by dealing with softer targets, namely actual MAP values.

Essentially, this study highlights the merit of the pattern recognition methodology as a whole in elucidating the existence of discriminatory information in the hemodynamic time series, rather than that of the trained classifiers as fixed models. When one prefers a different HE definition, data compilation is the only step that needs to be modified. With a new data set, a new classifier can easily be trained following the same subsequent steps.

### Pattern recognition performance

The classification and regression results in Tables 1, 2, 3, and 4 collectively indicate that multiple compilation led to more effective training data than single compilation. This finding implies that examples from the same record are not completely redundant and can be viewed as independent examples that encapsulate non-overlapping, discriminatory information. Given that single compilation can be seen as a random sample of multiple compilation, it can be inferred that single compilation was similar to using only a subset of available data for training, which is expected to result in weaker training. Further, this corroborates the fact that ICU data are generally non-stationary [10]. That is, the physiologic state of a patient varies over time during the same ICU stay, and hence data from different times in the same record should contain informative patterns. This kind of multiple compilation has been successfully applied to swallow segmentation with neural networks [29].

The promising classification and regression results based on multiple compilation (Tables 2 and 4) indicate that there exist discriminatory patterns in the data. However, accuracy should be interpreted with caution in such an unbalanced binary classification because favoring the majority class can easily guarantee a high accuracy. For example, there were up to 40 times as many control as hypotensive examples in multiple compilation. In this case, a classifier trained to blindly predict "control" all the time would achieve an accuracy over 97%. In comparison, AUC could be a more reliable performance measure, since it reflects a compromise between sensitivity and false alarm rate. Hence, the mean AUCs over 90% in Table 2 represent excellent overall classification performance. Bradley [30] has discussed the desirable properties of AUC as a classification performance metric in comparison with the conventional accuracy, stating that AUC is threshold-independent and invariant to *a priori *class probabilities.

On the other hand, the PPVs in Table 2 indicate that only about 1 in 8 positives is expected to be true at best. Since the corresponding sensitivities in Table 2 are reasonably high, we infer that the primary cause of the low PPVs is the overwhelming imbalance between the two classes. It should be noted, however, that the prominent class mismatch from multiple compilation reveals the true prevalence of HEs in real-life ICUs. Low PPVs originating from low prior probabilities of positive cases are a major problem in many classification problems, and low PPVs have been related to increased latency in human response to alerts [31].

### Utility as a hypotensive risk stratifier

Due to the low PPVs, the binary classifiers would have little clinical value as a direct HE predictor or "alarm". However, the clinical impact of the low PPVs can be greatly mitigated if the pattern recognition algorithms function as a hypotensive risk stratifier rather than an HE predictor. As mentioned in the Introduction section, there is considerable clinical value in identifying a subgroup of ICU patients with higher risk of developing an HE in the near future. The flagged patients would not necessarily need immediate intervention (such as vasopressor therapy), but rather increased vigilance would be appropriate. One way to implement such a risk stratifier based on the algorithms developed in this study is to flag a fixed number of patients in the ICU who exhibit the highest risk of hypotension. A trained pattern recognition algorithm from this study would continuously monitor each patient independently, but the risk stratifier would monitor the ICU as a whole and suggest which patients deserve the closest attention.

### Continuous monitoring

**Figure 2:**
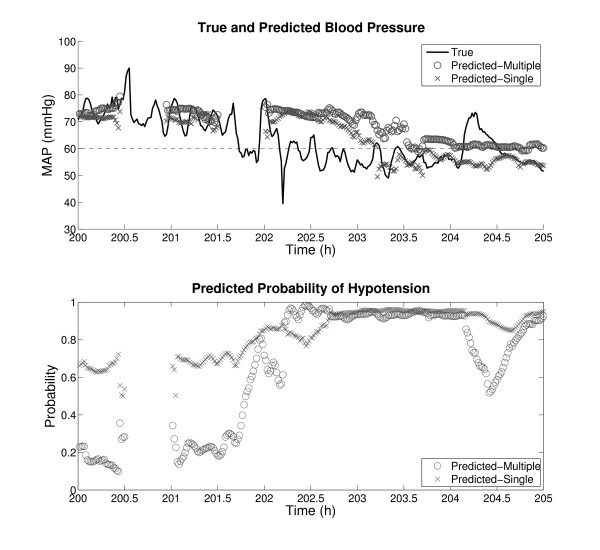
**An example of minute-by-minute continuous prediction with HEs**. The gap and observation window sizes are 1 and 0.5 hour, respectively. The top plot shows the true and predicted (from regression) MAPs, and the dashed line at 60 mmHg represents the HE threshold. The bottom plot shows the corresponding posterior probabilities of hypotension from the binary classifier. The results from both single and multiple compilation are shown. The periods of no prediction are due to inadequate input data. To facilitate comparison in the top plot, the predicted values were shifted to remove the gap. This implies that the predictions were actually generated 1 hour in advance. However, the bottom plot was not temporally aligned to preserve the predictive nature.

**Figure 3:**
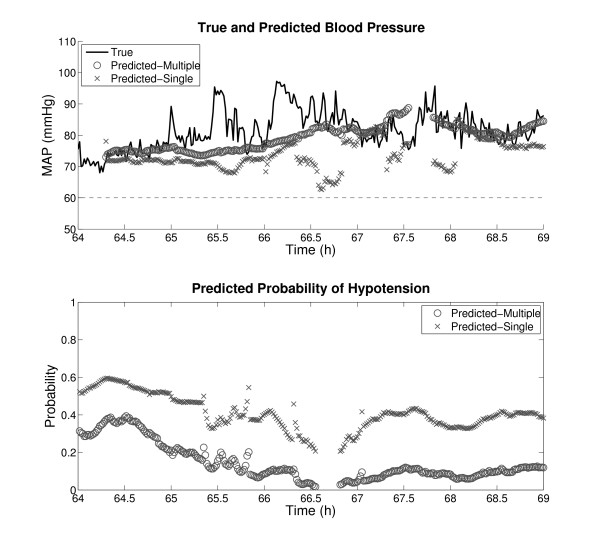
**An example of minute-by-minute continuous prediction with no HE**. For details, please see the Figure 2 caption.

One exciting aspect about the results in Tables 2 and 4 is that they were obtained from serially compiled examples that comprise records in entirety. This kind of data preparation simulates real-time continuous monitoring, and it is expected that comparable performance can be achieved in a real ICU. Continuous monitoring is often an integral part of clinical decision making, as has been shown in glucose monitoring [32] and EEG monitoring in the ICU [33]. In order to visualize how the classification and regression algorithms of this study performed in continuous monitoring, Figures 2 and 3 were generated as examples. Predictions were made every minute with a 1 hour gap and a 30 minute observation window. Figure 2 contains HEs, whereas Figure 3 does not. The periods of no prediction in both figures were caused by missing or out-of-physiologic-bound input data.

To facilitate comparison, the predicted blood pressures in the top plots of Figures 2 and 3 were shifted by one hour to account for the gap. However, no such temporal alignment was done in the bottom plots in order to preserve the predictive nature. Also, the reader is reminded that the predicted MAPs and probabilities of hypotension apply to 30 minute target windows, not individual minutes. In particular, the regression algorithm was trained to forecast the median MAP in the target window, which caused the smoothing effect on the predicted blood pressures in Figures 2 and 3.

These plots, especially Figure 3, once again support the finding that multiple compilation resulted in better performance than single compilation. In Figure 2, multiple compilation resulted in lower probabilities of hypotension than single compilation between 200 and 202 hours, exhibiting a more drastic difference between the normotensive and hypotensive regions. In Figure 3, multiple compilation shows better regression results and lower probabilities of hypotension than single compilation. Nonetheless, note that the predictions from multiple compilation are far from perfection. For instance, in Figure 2, not every 30 minute window in the hypotensive region roughly after 203 hours is an HE. Also, in both Figures 2 and 3, there are times when regression results substantially deviate from true MAPs. These observations again corroborate that a direct HE predictor would be disruptive in the ICU.

### Discriminatory information and physiology

**Figure 4:**
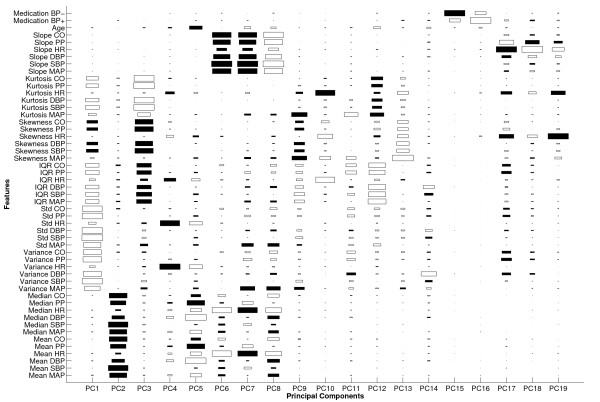
**A visual illustration of PCA loading on the first 19 principal components that captured 90% of the total variance in the entire multiple compilation data**. Only the statistical and clinical features are shown; see Figure 5 for the cross-correlation and wavelet features. The principal components are sorted in decreasing order of eigenvalue, with PC1 associated with the largest eigenvalue. The gap and observation window were 1 hour and 30 minutes long, respectively. The size of each rectangle is directly proportional to the magnitude of the loading. Filled and unfilled rectangles correspond to positive and negative loading coefficients, respectively.

**Figure 5:**
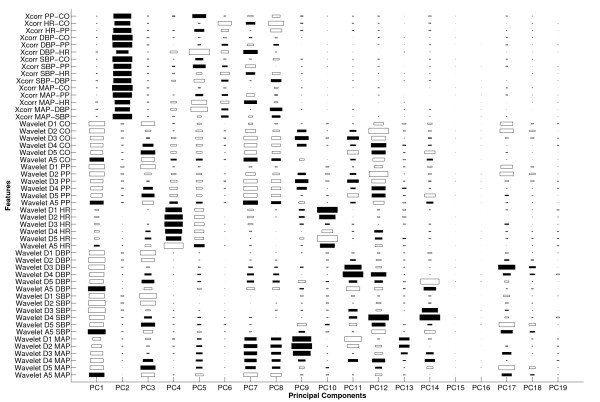
**Same visualization of PCA loading as Figure 4 for the cross-correlation and wavelet features**. See Figure 4 caption for more details.

Establishing a connection between the discriminatory features and physiology is a non-trivial task since machine learning finds discriminatory information from a purely mathematical perspective. The nonlinearity of the neural network and multi-dimensional feature space make the physiological interpretation even more challenging. Nevertheless, an essential step in this endeavor would be to scrutinize the PCA results. Although PCA is optimized to capture variance rather than discrimination, the promising classification and regression performance in this study implies that PCA successfully captured discriminatory information as well. Figures 4 and 5 provide a useful visualization of the feature contributions to the principal components with the 19 largest eigenvalues that captured 90% of the total variance in the entire multiple compilation data. Figure 4 shows the statistical and clinical features, whereas Figure 5 illustrates the cross-correlation and wavelet features. The actual PCA loading behind the results in Tables 1, 2, 3, and 4 were slightly different from Figures 4 and 5 because PCA was conducted for different training data sets (due to cross-validation and sub-sampling) to generate those results. The size of each rectangle corresponds to the magnitude of loading, whereas the filled and unfilled rectangles represent positive and negative loading coefficients, respectively.

The first two principal components mostly capture variance and magnitude information, respectively, suggesting that the dynamics and magnitude of the examined physiologic variables in the observation window are effective indicators of impending HEs. In addition, it is noticeable in the first two principal components that the contribution of HR is much less than that of the other 5 time series, while the fourth principal component mainly captures HR information. This suggests that HR is independent from the other physiologic variables which are mathematically correlated as a group. It is also worth noting that age and hemodynamically-active medications accounted for little variance in the data. Thus, it is inferred that perhaps only two time series, i.e., HR and one of the remaining 5 time series, might capture most of the non-redundant variance in the data and lead to comparable pattern recognition performance.

### Signal quality

One limitation of this research work is that no metric of signal quality was incorporated. Although crude upper and lower bounds based on physiologic feasibility were utilized, they certainly did not completely exclude all examples with artifacts and corruption. As a result, such unwanted components of the data may have clouded truly discriminatory information, confusing the neural networks during training. Previous research efforts have developed quantitative signal quality indices (SQI) for ECG [34] and ABP [35]. Since the time series utilized in this study were computed based on ECG and ABP signals, the ECG and ABP SQIs may well be employed as indirect SQIs.

## Conclusions

This work investigated the existence of discriminatory patterns in ICU data that can be indicative of impending HEs. The search for the discriminatory information was conducted via artificial neural networks. The binary classification (normotensive vs. hypotensive) and regression (prediction of actual MAP) performance confirmed that discriminatory patterns exist. By utilizing multiple examples from the same record (multiple compilation), promising predictive performance was achieved 1 hour in advance by observing the past 30 minute data. Poor PPVs arising from low HE prevalence limits the utility of the trained algorithms as direct HE predictors. However, a hypotensive risk stratifier based on the pattern recognition algorithms stemming from this study can mitigate adverse effects of the low PPVs. Such risk stratification would be of significant clinical value in busy ICU environments. The immediate future work is to implement and evaluate a hypotensive risk stratifier in a real-time clinical trial.

## Competing interests

The authors declare that they have no competing interests.

## Authors' contributions

JL designed the study, compiled the data, trained and evaluated the artificial neural networks, and drafted and revised the manuscript. RM participated in the design of the study and revised the manuscript. All authors read and approved the final manuscript.
